# Effects of Exergame Play on EF in Children and Adolescents at a Summer Camp for Low Income Youth

**DOI:** 10.5539/jedp.v4n1p209

**Published:** 2014-04-23

**Authors:** Rachel M. Flynn, Rebekah A. Richert, Amanda E. Staiano, Ellen Wartella, Sandra L. Calvert

**Affiliations:** 1 University of California, Riverside, USA; 2 Pennington Biomedical Research Center, USA; 3 Northwestern University, USA; 4 Georgetown University, USA

**Keywords:** adolescents, executive function, exergames, extracurricular activity, physical activity, video games

## Abstract

Past research has suggested exergame play improves adolescents’ executive function (EF) skills. EF change in 70 African American and Hispanic/Latino 10- to 16-year-olds participating in an inner-city summer camp was assessed following five 30-minute exergame play sessions. Children’s EF scores improved from pre- to posttest, and factors related to this change were examined. The number of exergame sessions the participants attended predicted posttest scores. In addition, level of achievement during game play was related to EF scores. Finally, the children’s level of enjoyment was not related to EF; however, frustration and boredom during game play were negatively related to EF. The findings are discussed in terms of their implications for the relationship between exergame play and cognitive benefits for adolescent players.

## 1. Introduction

Exergaming is a popular activity for youth that combines video gaming with physical activity (Staiano & Calvert, 2011). Engagement with exergames may impact the development and use of executive functioning (EF) skills, which include attention, inhibition, and memory (Best, 2010; Staiano, Abraham, & Calvert, 2012a). EF skills work together in higher-order cognitive functions such as problem-solving (Zelazo, Carter, Reznick, & Frye, 1997) and are related to math and reading abilities, even after controlling for general intelligence and classroom behavior (Blair & Razza, 2007; Riggs, Blair, & Greenberg, 2004). Research has revealed a range of early experiences can influence the development of these skills (Best, 2010; Zelazo et al., 1997). Past studies have indicated some benefits to EF skills after playing exergames (Best, 2012; Staiano et al., 2012a). Although recent research has explored the contexts and interventions, such as exercise, that can boost EF in children and adolescents (Diamond & Lee, 2011), little is known about the mechanisms influencing EF gains from exergame play.

The current study examined using an exercise-based video game (i.e., exergames) to increase EF skills in a diverse sample of youth from a low-income neighborhood in the South Bronx in New York City, a population with high rates of childhood obesity (NYCDOHMH, 2006) and low EF (Raver, Blair, & Willoughby, 2013), who were in need of sustainable physical activity interventions. The goal of the current study was to examine the influence of playing exergames on children’s EF skills with a particular interest in individual factors that might contribute to growth in EF, such as enjoyment and engagement in playing the game, level of achievement/difficulty in game play, and previous video game experience.

### 1.1 Exergames as a Form of Exercise

Exergames include any video game that involves gross motor skills (Staiano & Calvert, 2011). There is a wide range of interactivity within the genre, from arm and hand movements (e.g., Nintendo *Wii Sports* games such as boxing, bowling and tennis) to full body movements (e.g., Nintendo *Wii Fit*, Nintendo *Wii Active*, Microsoft *Kinect*, and Konami *Dance Dance Revolution*). These games are popular with youth: a 2011 report of game sales showed that six exergames were in the top 20 best-selling games since 1995 (Walton & Mazel, 2011). When children play exergames, they expend energy at a moderate rate, as measured by accelerometers, heart rate monitors, and expired breath analyzers (Biddiss & Irwin, 2010; Daley, 2009; Peng, Lin, & Crouse, 2011). Exergames that use the entire body, such as the Nintendo *Wii Fit*, promote significantly higher energy expenditure than sedentary video games or even mildly-active video games such as the Nintendo *Wii Sports* (Graves, Stratton, & Ridgers, 2008; Graves, Ridgers, & Stratton, 2008; Lanningham-Foster et al., 2006; Lanningham-Foster et al., 2009).

Several studies have found that energy expenditure while playing exergames that use the whole body is equivalent to moderate physical activity (Graf, Pratt, Hester, & Short, 2009; Mhurchu et al., 2012; Prapavessis, & Rodger, 2008). Graf et al. (2009) found that playing Konami *Dance Dance Revolution* (DDR) on Level 2 and boxing and bowling in Nintendo *Wii Sports* had similar or higher energy expenditure and heart rates when compared to walking moderately on a treadmill. Thus, exergames are a viable source of physical activity for children, implying these games can be compared to exercise when used with the goal of promoting fitness. Indeed, the Compendium of Physical Activities now lists the exergame Nintendo *Wii Fit* as a conditioning activity (Ainsworth et al., 2011). Additionally, children enjoy exergames as a form of physical activity: children participating in a physical education class that incorporated exergames for six weeks improved their attitudes toward physical activity more than those participating in physical education without exergaming (Lwin & Malik, 2012).

### 1.2 Cognitive Benefits of Exercise and Video Game Play

Exercise not only produces physical benefits, but can improve many aspects of cognition during childhood, including EF and academic performance (Hillman, Erickson, & Kramer, 2008; Tomporowski, 2003). According to a meta-analysis of 44 studies totaling 125 effect sizes, physical activity was related to improvements in cognition, including perceptual skills and academic outcomes, for children and adolescents ranging from 4 to 18 years old (Sibley & Etnier, 2003). In other studies, aerobic activity interventions improved EF for 7- to 11-year-olds immediately after a 10- to 40-minute session of physical activity when compared to control groups (Ellemberg & St-Louis-Deschênes, 2010; Hillman et al., 2009). Researchers have hypothesized that participation in physical activity, exercise and sports requires complex and flexible cognitive skills (Tomporowski, Davis, Miller & Naglieri, 2007). For example, when playing a sport or game, children work with teammates toward a common goal while using strategies and rules. Aerobic exercise particularly requires adapting and modifying physical movements in a goal-directed manner. Executive functioning skills are required for this type of task planning and monitoring (Tomporowski et al., 2007).

Playing interactive video games is a goal-directed activity that requires memory, attention, knowledge, and problem solving (Greenfield, 1996). Because using interactive media requires visual perception, fine motor skills, and selective attention, the use of these tools influences children’s development of these cognitive skills (Blumberg, Altschuler, Almonte, & Mileaf, 2013; Calvert, 2004; Gauvain, 2001; Greenfield, 1996). Video games require making decisions in a fast-paced, changing environment that often involves mentally rotating objects or other spatial orientation skills. Given the structure of these games, it is unclear if the improvements in cognition post-video game play are due to the nature of the video game or the fact that the player is cognitively engaged in an activity he or she enjoys (Green & Bavelier, 2007).

According to Rideout, Foehr, and Roberts (2010), video game play is an increasingly common experience that shapes children’s developmental landscape. Over 90% of children between the ages of 6 and 18 have played video games, and 60% of children aged 8 to 18 play video games an average of one hour a day (Rideout et al., 2010). Studies have found that children ages 8 to 11 can develop visual attention, spatial relations, and mental rotation from playing interactive games (Bottino, Ferlino, Ott, & Tavella, 2007; De Lisi & Wolford, 2002; Subrahmanyam & Greenfield, 1994). Computerized training studies using games designed specifically to improve EF skills for children have been effective in both the short and long term (Thorell, Lindqvist, Nutley, Bohlin, & Klingberg, 2009). Additionally, playing video games improved cognitive strategy skills, cognitive flexibility, spatial relations, reasoning, and visual attention in adults (Bavelier et al., 2011; Feng, Spence, & Pratt, 2007).

### 1.3 Exergames: Combining Exercise and Video Game Play for EF Skills

Exergames have the potential to have a significant impact on EF skills based on the combination of exercise and cognitively-engaging video game play (Anderson-Hanley, Tureck, & Schneiderman, 2011; Anderson-Hanley et al., 2012; Best 2010; 2012). This increased interactivity has several potential benefits related to the physical activity required during game play and the cognitive benefits of combining gross motor skills and the cognitive engagement of video game play. By combining physical activity with game play, exergames may influence EF more strongly than traditional gaming or physical activity alone (Anderson-Hanley et al., 2012; Best, 2010; Staiano et al., 2012a). Anderson-Hanley et al. (2012) found that exergaming provided cognitive benefits beyond the benefits of exercise in a sample of older adults. Gao and Mandryk (2012) found that 10 minutes of exergame play had an acute positive impact on EF for adults.

In contrast to the studies conducted with adults, few studies have examined the influence of exergames on children’s EF skills (Green & Bavelier, 2007). In one study on exergames and EF, adolescents who played an exergame competitively against a peer improved EF more than an exergame group that played cooperatively with a peer or a no exergame exposure control group (Staiano et al., 2012a). In Staiano et al., 15- to 19-year-old African American adolescents participated in a 10-week fitness intervention program. The majority of the children were overweight or obese and from a low-income neighborhood. There were three conditions: a competitive exergame group, a cooperative exergame group, and a control group, which played a non-active video game. Participants in the competitive exergame condition improved more than adolescents in the control condition though there were no significant differences between the adolescents in the cooperative and control conditions (Staiano et al., 2012a). The findings suggested adding competition during exergame play led to greater EF improvements, perhaps because youth might be more engaged or more challenged during this type of play.

Anderson-Hanley et al. (2011) conducted a study of cognitive improvements comparing participants in a DDR condition to participants in a video-watching control condition. Children in the study were ages 10 to 18 and diagnosed with an autism spectrum disorder. Performance on the Digits Backwards task, a measure of EF, improved more for children in the exergame condition than the video-watching condition (Anderson-Hanley et al., 2011). In a within-subjects study, Best (2012) found that 6- to 10-year-old children’s level of physical activity, but not cognitive engagement, while playing an exergame was related to increased ability to resolve interference from conflicting stimuli. Each child participated in a 20-minute activity on four different occasions: a video watching session, a sedentary video game session, a jogging exergame session, and an aerobic obstacle course exergame session. Children reported being more engaged while playing the sedentary video game and the obstacle course exergame than while watching the video and playing the jogging exergame (Best, 2012). In addition, while watching the video game and playing the jogging exergame, children verbalized more frustration and enjoyment than while participating in the other two sessions (Best, 2012). However, EF skills were higher immediately after play for the two exergame sessions compared to the sedentary sessions. There were no differences in EF after the cognitively-engaging (i.e. sedentary video game and aerobic exergame) sessions compared to the non-cognitively engaging (i.e. video watching and jogging exergame), suggesting exercise may have a stronger influence on EF than engagement and enjoyment while doing an activity.

### 1.4 The Current Study and Hypotheses

Although past research has suggested exergames have the potential to improve EF, the specific factors that can lead to improved EF skills are unclear (Diamond, 2012). Therefore, the goal of the current study was to examine the individual difference factors that might contribute to adolescents’ improvement in EF after playing an exergame.

Based on previous research (Davis et al., 2011; Diamond, 2012; Diamond & Lee, 2011; Greenfield, 1996; Staiano et al., 2012a), the hypotheses were that children’s level of improvement in EF skills after playing a highly-active exergame would be positively related to: (a) their prior experience with exergames, as those children with experience may be able to challenge themselves more than those who are first learning the game; (b) their game play expertise, which could result in higher levels of achievement of game play and challenge, leading to improved EF skills, (c) the number of times the game is played, an indicator of current exposure; and (d) their level of engagement while playing the game, an indicator of motivation (Diamond & Lee, 2011; Greenfield, 1996). In addition, if exergame play impacts EF skills, then children who regularly play exergames may begin the study with higher EF skills than those who do not play exergames (e.g., Greenfield, Dewinstanley, Kilpatrick, & Kayne, 1994).

## 2. Method

### 2.1 Participants

The participants in the Wii Fit group were 70 10- to 16-year-old children (*M* = 13.72 years, *SD* = 1.41; 48% female). In terms of ethnicity, 48% were African American, 47% were Hispanic/Latino, and 5% were biracial. The research took place at a six-week summer camp for low-income students from neighborhoods surrounding the South Bronx, NY. Parents provided consent, and children provided assent before the study began. Children, families and camp staff were informed that children could withdraw from the study at any point during the summer. An Institutional Review Board approved all procedures and materials. Within four age groups (i.e., 9- to 10-year-olds, 11- to 12-year-olds, 12- to 13-year-olds, and 14 years and older), the camp director randomly assigned participants to groups of 15 children.

### 2.2 Materials

#### 2.2.1 Nintendo Wii Fit (Note 1)

Wii Fit is an exercise-based game that requires controlled movements. Players must maintain an active physical motion with balance while standing on the balance board. Players hold the Wiimote controller while playing, and some games require arm movements with the Wiimote. The Wiimote controller and balance board transmit a player’s motion to a sensor bar placed on top of the television.

#### 2.2.2 Nintendo Games

Children played aerobics games and balance games, including hula hoop, step aerobics and jogging on the Wii Fit. The games were accompanied by music and had audio and visual instructions and cues throughout the exercise. The games provided progress screens at the end of each exercise. Progress was tracked with level achieved (e.g. number of stars or points accrued) and fitness achievement (e.g. calories burned). For example, after the hula hoop game a screen would show the text “Your score: 245 spins. 2 stars.” The children played five games at each weekly session: Step Aerobics, Hula Hoop, Table Tilt, and two additional games from the Wii Fit package, which rotated weekly so children played all the available games. These games were chosen based on evidence that short exergame sessions could improve EF for adolescents (Staiano et al., 2012a). All games were rated “E” for everyone.

### 2.3 Procedure

A trained researcher who had a Masters degree in physical education and was a licensed teacher by the State of New York led each session. During the first session, the researcher administered the pretest D-KEFS in a group setting; children completed the demographic and media questionnaire, and the participants had a shortened session of video game play. For the following four weeks, participants attended an hour-long session once a week. In week six, all youth were tested on the EF measures.

As part of their overall summer camp experience children participated in swimming, sports, fine arts, performing arts and technology activities. Children participated in four of these summer camp activities daily from 9:00 am to 5:00 pm with their assigned camp group. There were 30 different summer camp activities, and each group had 20 different activity sessions a week. Children participated in the research study with their camp group on the same day and time once each week. Only one group (of 15 children) participated in the research study at a time. Each hour-long session took place in a classroom with eight stations equipped with a flat screen 32-inch television, Nintendo Wii game console, two Wiimotes, and a Wii Fit balance board. Camp counselors accompanied the children to the sessions and were available to help children complete the surveys (e.g., by clarifying questions) and to accompany children to the restroom if needed. Two children were randomly assigned to a station. Some of the dyads were same-gender and some were mixed-gender. At each station, one child played at a time while the second child observed or helped record scores. The sessions began with the participant selecting her or his virtual character. Then children followed the specific exercise routine and recorded their progress on their exercise log. Each child played the games for a 30-minute session and then filled out post-playing surveys.

### 2.4 Measures

#### 2.4.1 Delis-Kaplan Executive Function System (D-KEFS)

EF skills were tested using the pencil-and-paper *Delis-Kaplan Executive Function System (D-KEFS)*, which was the same method used in a previous study demonstrating improved EF skills after exergame play (Staiano et al, 2012a). Because of the kinds of cognitive skills that have improved after traditional video game play (e.g. visual-spatial skills, visual acuity, visualization, task switching, and perceptual speed), we administered the Design Fluency subscale of the *D-*KEFS, which requires higher EF skills and underlying component skills (Delis, Kaplan, & Kramer, 2001). The Design Fluency subscale measures visual attention, response inhibition, planning, motor speed, and cognitive flexibility (Delis et al., 2001).

The goal of the Design Fluency scale is to connect dots to create novel shapes as quickly as possible in 60 seconds. The participant must connect four lines to create a different design in each of the 35 boxes on the subscale. There are three design subscales: (a) Design 1 Filled Dots; (b) Design 2 Empty Dots; and (c) Design 3 Switching Dots. Design 1 tests fluency. In this subscale, all dots in the box are solid and the participant may connect any dots to create novel shapes. Design 2 tests fluency, response inhibition, motor planning and speed. In this subscale, the box contains 5 solid dots and 5 empty dots; the participant must only connect empty dots to create novel shapes. Design 3 tests fluency, visual-attention, visual scanning and cognitive flexibility. This design is the most complicated as the box contains 5 solid dots and 5 empty dots. The participant must alternate in connecting a solid and empty dot to create novel shapes. The total score for each design subtest is total number of shapes correctly drawn, with a maximum possible score of 35. The total possible score on the Design Fluency scale was 105. Scaled scores were computed from the raw scores using the normative scores by age group and ranged from 1 to 19 (Cronbach’s *α* = .90).

#### 2.4.2 Number of Wii Fit Sessions

Participants were scheduled to participate in the study weekly for six weeks with their camp group. The pretest and a shortened familiarization game session occurred in the first week, the following four weeks were game sessions, and the sixth week was the posttest. The trained researcher recorded the number of times each child played. Children who were absent the day their group attended the session or feeling too ill to fully participate were not able to make-up their Wii Fit session, based on the camp’s schedule. The variable *Number of Sessions* was created by adding the total number of times each child played the Wii Fit. On average, participants attended 3.90 (*SD* = 1.20) out of 5 possible Wii Fit classes: 42% participated five days, 19% participated four days, 21% participated three days, 14% participated two days, and 4% participated one day.

#### 2.4.3 Media Exposure Questionnaire

Participants completed a media exposure questionnaire examining media use for children (Rideout, Vandewater, Wartella, & Kaiser Family Foundation, 2003). In pretest, participants indicated if they had ever played Wii Fit or any Wii games. A “no” response was scored as 0. If they had used either type of media, children were asked, “How often do you play?” Participants responded to the questions on a five-point scale including the following response categories: *almost every day* (5), *a few times a week* (4), *about once a week* (3), *every now and then* (2), and *just once or twice* (1). A *Frequency of Game* play variable was created by selecting the highest score on either of these of these two variables (range = 0 to 5), with scores of 5 indicating the participant played either type of Wii game a few times a week and a score of 0 indicating the participant did not play either type of Wii game.

#### 2.4.4 Level of Engagement

To measure children’s engagement during Wii Fit play, children responded to three affective reaction questions indicating their enjoyment, boredom, and frustration. In posttest, participants were asked, “How much did you enjoy playing Wii Fit this summer?” Participants responded to the question on a 4-point scale (which constituted their *enjoyment* score): *I really like playing* (3), *I sort of like playing* (2), *I sort of don’t like playing* (1), *I really don’t like playing* (0). Participants were also asked to rate their agreement with two additional statements: “the game was boring to play” and “the game was frustrating to play.” Participants responded to these statements on a 5-point scale for what was true for them: *strongly disagree* (1), *somewhat disagree* (2), *neutral* (3), *somewhat agree* (4), *strongly agree* (5). Responses to these two questions comprised the *boredom* and *frustration* scores, each of which ranged from 1 to 5.

#### 2.4.5 Level of Achievement

Participants tracked their game play by recording the number of levels achieved in each game on a weekly log. Under the supervision of the research assistant and camp counselors, the participants recorded their progress in an exercise log in order to track week-to-week improvement. The level achieved is related to the amount of physical activity during game play. For example, a higher level of achievement indicates more steps taken with perfect timing or more times spinning the hula-hoop. The average level of proficiency could range from 0 (lowest) to 5 (highest). In order to assess *level of achievement*, the average level achieved across games was calculated for each participant in the first (*M =* 1.35, *SD* = .53, range = 0 to 5) and last week (*M* = 1.73, *SD* = .69, range = 0 to 5) he or she participated.

#### 2.4.6 Body Mass Index

The trained researcher measured the height and weight of each child. Weight was measured using a Tanita scale with the participant fully clothed, but not wearing shoes. Height was measured using a standard measuring tape. Body mass index (BMI) was calculated using the Center for Disease Control’s (CDC) formula, weight in pounds (lb.) divided by squared height in inches (in) and then multiplied by the conversion factor of 703. BMI was then converted to a percentile based on gender and age as determined by CDC growth curves for ages 2 to 20 years. A percentile under 85% is considered normal weight, a percentile between 85% and 95% is considered overweight, and a percentile above 95% is considered obese (Kuczmarski et al., 2000). About 63% of participants were normal weight, 20% were overweight, and 16% were obese.

### 2.5 Control Participants

Two control conditions were conducted with a limited sample from the camp in order to examine general trends in participants’ change on the D-KEFS from pre- to posttest. One group of participants played a less active exergame called Boomblox (*n* = 10) and one group was a non-playing control (*n* = 14). The researcher randomly assigned the groups to these conditions, and the sample sizes for these groups was controlled by the camp administrators, who wanted as many children as possible to participate in the Wii Fit sessions.

The same protocol was followed as the procedure described above. Every child at camp had a daily sports activity. Children in the Boomblox group played the video game as one of their five daily sports activities, and children in the non-playing control group had five regular sports sessions. Participants in the non-playing control group completed the pre- and posttests of the D-KEFS during one of the times reserved for a sports activity and did not attend a weekly session. Participants in the less active exergame group played the game Boomblox, a puzzle action game on the Wii console. In this game, the goal is to throw an object at a pile of stacked blocks in order to knock the blocks down. Different levels have different challenges, such as to only hit the blue blocks, to use extra speed on the throw or to destroy the tower as quickly as possible. The player used the Wiimote and performed a throwing action similar to pitching a softball to virtually “throw” the object at the blocks.

#### 2.5.1 Statistical Analysis

Preliminary analysis explored differences in demographic characteristics (e.g., age, gender, ethnicity, session attendance, BMI, prior game exposure) by group (see Table 1). Chi-square analyses revealed there were no significant differences by condition in gender, ethnicity, BMI, or prior game exposure.

The small sample sizes in the two control groups (i.e. less active exergame and non-playing control) limited the ability to conduct statistical analyses comparing children in these two groups to the children in the Wii Fit group. Therefore, analyses of the two control groups focused on exploring the degree of pretest to posttest change. Paired samples *t*-tests were used to examine if participants increased or decreased from pretest to posttest on the scaled design subscale scores. The Boomblox group improved from pretest (*M* = 4.70, *SD* = 4.22) to posttest (*M* = 7.40, *SD* = 6.82), but not significantly. Additionally, there were no significant differences from pre- (*M* = 8.57, *SD* = 6.81) to posttest (*M* = 8.57, *SD* = 4.94) within the non-playing control group condition. These findings suggest that if participants in the Wii Fit condition demonstrate increases in EF skills, that these findings can be attributed to playing the Wii Fit for five weeks rather than having completed the EF measure twice. Further analyses were conducted on the Wii Fit group only given the small sample sizes in the control groups.

## 3. Results

The analyses were conducted on the Wii Fit group only. The first set of analyses examined whether there was significant improvement in the participants’ EF scores after five weeks of exergame play. The second set of analyses examined the relationships between participants’ EF scores and the number of sessions they attended, as well as their levels of achievement and engagement while playing the exergrame. The final set of analyses examined whether any of the individual difference factors predicted participants’ EF improvements while controlling for pretest.

### 3.1 Change in EF Scores

**Figure 1:**
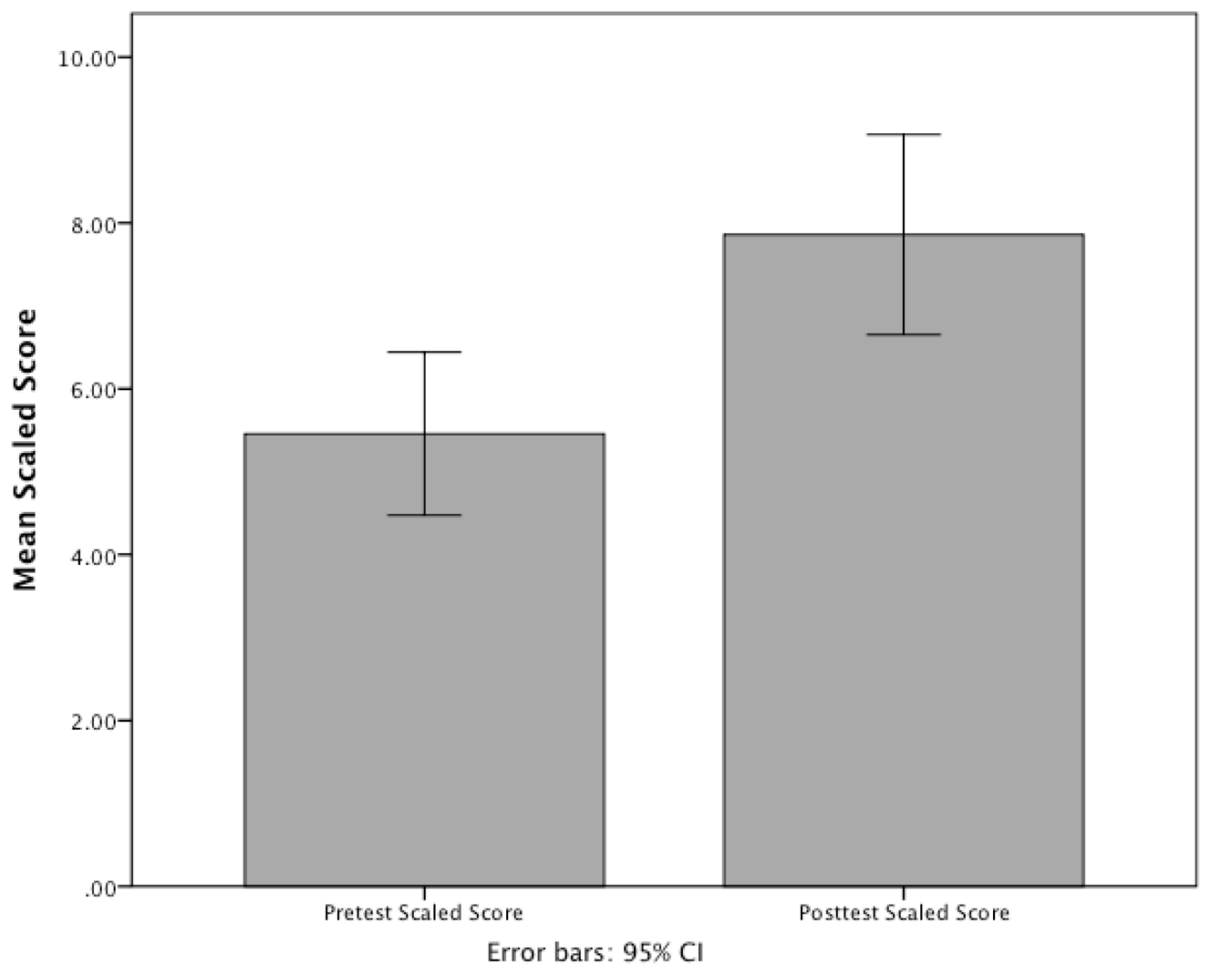
Pretest and posttest scaled EF scores of the exergame group

Children who played the exergame scored between 0 and 60 (*M* = 12.73 *SD* = 11) on pretest and between 0 and 62 (*M* = 19.33, *SD* = 14.92) on posttest, out of a total possible 105 points on the Design subscale. The scaled scores ranged from 1 to 19, averaging 5.45 (*SD* = 4.12) in pretest and 7.85 in posttest (*SD* = 5.06). All analyses were conducted using the normed-scaled scores, therefore age was not included as a covariate in any analysis. As is depicted in Figure 1, paired samples *t-*tests revealed that the participants did significantly better in posttest than in pretest assessments of EF, *t*(69) = 4.32, *p* < 0.001. Based on the improvement demonstrated by participants who played the exergames, further analyses examined individual difference factors related to children’s improved EF scores over the five-week intervention. Independent samples *t*-tests were used to examine whether pre- and posttest EF scores significantly differed by gender. There was not a significant gender difference at pretest or at posttest.

### 3.2 Individual Difference Factors: Relationships with EF Change

Bivariate correlational analyses were used to examine the relationship between pre- and posttest EF scores and individual difference factors. There was a significant positive relationship between pretest and posttest EF scores, *r* = .50, *p* < .001. Participants’ pre- and post-EF scores were significantly correlated with the number of exergame sessions the participants attended, as well as the participants’ level of achievement (see Table 2). The more sessions the participants attended, the higher their posttest EF score. Children with higher pretest EF scores also achieved higher levels of game play, and higher levels of game play were correlated with higher posttest EF scores. Finally, there was a significant negative relationship with pre- and posttest EF scores and frustration and boredom (see Table 2). Children who were more frustrated or bored had significantly lower pre- and posttest EF scores. There were not significant relationships between enjoyment or BMI and pre- or posttest EF scores.

The relationship between children’s EF improvements and previous exposure to playing Wii Fit or other Wii games was examined. About 50% of children had played Wii Fit before, and 71% of children had played a game on the Wii before. Correlational analysis revealed that children who had previously played Wii Fit more often achieved higher levels in the game, *r* = .26, *p* < .05; however, there was no significant correlation between amount of prior Wii play and pre- or posttest EF scores.

### 3.3 Regression Analyses

The correlational relationships between individual difference factors and EF scores justified the use of regression analyses. Therefore, regression analysis was conducted for each of the variables that related to posttest EF scores (Baron & Kenny, 1986). In all analyses, posttest EF score was the dependent variable, pretest EF score was entered in the first step, and the individual difference variable was entered in the second step. A simple regression analysis predicting posttest EF from pretest EF revealed that pretest EF scores significantly predicted posttest EF scores (*R^2^* = .24, *β* = .49, *p* < .001).

#### 3.3.1 Number of Sessions

First, we examined if number of sessions played was related to differences in EF scores. Pretest EF was unrelated to the number of exergame sessions participants played. A simple regression analysis predicting posttest scores from the number of exergame sessions, while controlling for pretest scores, indicated the number of sessions significantly predicted the posttest scores (see Table 3). The addition of number of sessions in the model accounted for an additional 7% (R Square Change) of the variance. These two predictors collectively accounted for 29% of the variance in posttest EF scores, and each remained significant when included in the same model.

#### 3.3.2 Level of Achievement

Next, we examined if game play achievement was related to individual differences in EF scores. Pretest EF scores were related to children’s level of achievement in the last week of playing the exergame. There was also a significant correlation between the level of achievement in the last week and the posttest EF score. A simple regression analysis revealed that pretest EF scores predicted level of game achievement in the last week, *R^2^* = .101, *β* = .32, *p* < .05. In addition, level of achievement in the last week predicted posttest EF scores *R^2^* = .085, *β* = .29, *p* < .05. A simple regression analysis predicting posttest scores from the level of achievement, while controlling for pretest scores, indicated that the level of achievement did not significantly predict the posttest score. The only significant predictor in the model was pretest (see Table 4).

#### 3.3.3 Engagement Factors

The final set of analyses examined whether there were differences in EF scores based on whether children enjoyed playing the game, found the game frustrating or found the game boring. In general, participants reported at the end of the program that they enjoyed playing the Wii Fit (*M* = 2.66, *SD* = .63, range = 0 to 3). Participants’ enjoyment was not related to pre- or posttest EF scores.

However, as reported in the correlational analysis above, there was a significant negative relationship with EF scores and frustration and boredom. Participants who rated the game as more boring or frustrating to play had significantly lower pretest and posttest EF scores. A simple regression analysis revealed that pretest EF scores negatively predicted reported level of frustration, *R^2^* = .085, *β* = −.29, *p* < .05. In addition, frustration negatively predicted posttest EF scores, *R^2^* = .09, *β* = −.30, *p* < .05. A simple regression analysis predicting posttest scores from the level of frustration, while controlling for pretest scores, indicated the level of frustration did not significantly predict the posttest score. The only significant predictor in the model was pretest (see Table 5).

Similar to participants’ reports of frustration, a simple regression analysis revealed that pretest EF scores negatively predicted reported level of boredom, *R^2^* = .101, *β* = −.32, *p* < .05. In addition, boredom negatively predicted posttest EF scores, *R^2^* = .221, *β* = −.47, *p* < .05. A simple regression analysis predicting posttest scores from the level of boredom, while controlling for pretest scores, indicated boredom significantly predicted the posttest scores (see Table 6). The addition of level of boredom in the model accounted for an additional 7% (R Square Change) of the variance. These two predictors collectively accounted for 52% of the variance in posttest EF scores, and each remained significant predictors or posttest EF when included in the same model.

## 4. Discussion

Few studies have explored the effects of playing exergames on cognitive outcomes (Staiano & Calvert, 2011), even though video games, especially the newest, physically-interactive technologies, are prevalent and popular activities for children and youth (Rideout et al., 2010). The present research extends previous research examining how exergame play impacts EF (Best, 2012; Staiano & Calvert, 2012a). To examine the influence of playing an exergame on EF skills, 10- to 16-year-old children participated in a Wii Fit study. Adolescents who played an exergame improved in EF over a five-week study, extending prior findings indicating that exergames were related to acute increases EF in adolescents (Staiano et al., 2012a; Best, 2012). In contrast, the non-playing control condition did not show improvement in EF scores from pre- to posttest.

Given the improvements in performance for the participants playing the exergame, further analysis considered individual difference factors related to the degree of improvement in EF performance. Prior experience playing video games did not influence EF scores. However, children’s level of achievement was related to EF scores; and the number of exergame sessions the participants attended predicted posttest scores. Finally, EF scores were partially related to children’s level of engagement while playing the game as measured by enjoyment, frustration, and boredom. Enjoyment was not related to EF, but frustration and boredom were negatively related to EF. We discuss each of these findings in turn below.

The first individual difference factor examined was the number of times the child played the exergame. Prior research has found cognitive benefits (e.g., improvement in cognitive strategies, cognitive flexibility, spatial relations, reasoning, and visual attention) from video game play in adults (Feng et al., 2007; Green & Bavelier, 2007). In the current study, children’s EF was more likely to improve if they played the exergame more, which extends past research documenting the short-term effects of gaming to improve EF skills (Staiano et al., 2012a).

The second individual difference factor that was considered in relation to the increase in EF skills for participants in the exergame group was prior exposure to video games. Greenfield, Brannon, and Lohr (1994) found prior video game experience significantly impacted pretest spatial skills, such that those with more experience had higher spatial skills than those with less experience. The researchers found that video game training over 10 weeks did not improve spatial skills for either group (Greenfield et al., 1994). In contrast to this past research, participants’ amount of prior game play experience was not significantly related to pretest EF skills. One possible reason for the difference in findings is that participants in Greenfield’s et al.’s (1994) study were undergraduates and those with video game experience were mostly male and reported playing about once a month. On average, children in the current study played any game on the Wii less than once a week, though most participants had video game experience. The differences between the findings in the current study and research with undergraduates highlights the need for further research on the developmental factors that influence the relationship between EF and video game play in early adolescence.

The third individual difference factor examined was level of achievement during game play. There was a significant positive correlation between pre- and posttest EF scores and the level of achievement during game play, indicating that children who achieved high levels of game play had higher EF scores in both pre- and posttest. These findings could reflect a number of mechanisms that may account for increases in EF skills after playing an exergame. First, children who already have higher EF skills may be better at exergames. In the current study, EF skills were related to achieving higher levels in game play, but those higher levels of play do not necessarily lead to greater growth in EF after game play. Given that scaffolding during video game play likely comes from the interactivity of the game itself (Dipietro et al., 2007), video games in general have the potential to provide sensitive scaffolds to children’s EF skills. From this perspective, if the game has the ability to target children in their zone of proximal development (Vygotsky, 1978), then children who are being challenged would benefit the most from exergame play. However, these children may not achieve as high a level of achievement as their peers, suggesting that playing at a level that challenges the child may be more important than the specific difficulty level that the children are able to achieve in playing the game.

Second, the relationship between level of achievement and EF skills may be because the children who played the exergame at a more difficult level could have invested more physical energy while playing. While not a direct measurement of physical exertion, levels of achievement were related to amount of physical activity in this exergame because all children completed each game and all the games had a set amount of time regardless of proficiency. For example, levels of achievement increased with the number of times a participant spun the hula-hoop or took a step. In this case, the physical engagement during game play would be the factor contributing to cognitive benefits. One recent study has suggested that cognitive engagement in video game play is not sufficient to explain effects of exergames on cognition, therefore physical activity may be accounting for the increase in EF skills (Best, 2012). Competing with the explanation that achievement level served as a proxy for physical exertion is the fact that a child who kept failing at a particular level may have exerted the same amount of physical effort as a child who succeeded throughout the level. Given the various explanations for the findings related to EF skills and level of achievement, future research should continue to examine the relationship between the level of difficulty of children’s exergame play and potential improvements in EF skills.

The final individual difference factor that was examined was children’s engagement in the activity. Participants’ rating of their enjoyment while playing the games was not significantly related to participants’ differences in EF scores from pretest to posttest; however, children reported enjoying the games for the most part. Thus, children’s levels of frustration and boredom while playing the games were also assessed. Frustration and boredom were significantly related to each other and to EF scores. Children with lower levels of EF experienced more frustration and boredom while playing the video game; however, only boredom was a significant predictor of posttest EF skills after controlling for pretest EF. In contrast, frustration was not significant when included in a model with pretest EF scores.

One possible reason for the different patterns in the regression models is that frustration and boredom reflect different patterns of engagement. More specifically, frustration may have indicated that children were actively engaged, but were frustrated by their level of achievement. In this case, although children were frustrated, they still improved in their EF scores as a result of playing the exergames. This interpretation is consistent with the finding that level of achievement itself did not predict gains in EF pre and postgame play, but that number of sessions of game play did predict EF gains. Children’s efforts while playing the exergame and their amount of exposure to the exergame both contributed to increases in EF scores.

In support of this interpretation, boredom likely indicates a lack of engagement with the game play. When children were bored and unengaged with the exergame activities, they did not demonstrate gains in EF. Importantly, most children enjoyed the game regardless of whether or not they achieved a high level. Although participants’ ratings of enjoyment did not seem to account for the pattern of increases in EF skills after playing an exergame; overall, almost all children reported they enjoyed the game. Therefore, exergame play in this applied setting was an enjoyable activity for children.

### 4.1 Implications

Exercise is beneficial to youth because it has physical benefits and also because it can improve EF skills (Best, 2010; Tomporowski et al., 2011). Therefore, activities and interventions that promote physical activity have multiple benefits. The findings from the current study have implications for understanding media use in adolescence. Media use is often assumed to be at the root of children’s inactivity (Marshall, Biddle, Gorley, Cameron, & Murdey, 2004). However, there are many factors that might cause children to be characterized as sedentary, meaning they get less physical activity than recommended, such as lack of safe outdoor spaces, reduced physical education time and lack of access to enjoyable sports programs (Biddle, Gorely, Marshall, Murdey, & Cameron, 2004; Lindsay, Sussner, Kim, & Gortmaker, 2006). Two of the most cited barriers to physical activity are lack of enjoyable options or lack of time (Tergerson & King, 2002).

Interventions that increase physical activity, such as exergame play, are of interest as the rates of childhood obesity in the U.S. tripled over the last 40 years (Anderson & Butcher, 2006; Ogden, Carroll, Kit, & Flegal, 2012). Nationwide, African-American and Hispanic children, as well as low-income children, are more at risk for obesity than their white middle class counterparts (Anderson & Butcher, 2006; Durant, 2009; Kumanyika & Grier, 2006). In addition, Rideout et al. (2010) found that Black and Hispanic youth consume media at a significantly higher rate (13 hours daily) than their Caucasian peers (8.5 hours daily), even after controlling for age, parental education, and single family homes. Most of this difference is composed of television viewing; however, Black and Hispanic youth also spend 30 minutes more daily playing video games.

Diamond and Lee’s (2011) review of research stated that interventions and activities must be sustainable if they are to improve EF; therefore, video games must be enjoyable and engaging enough to holding children’s attention over time. Exergames have the potential to promote physical activity in schools and community settings by capitalizing on the popularity of video game play (Staiano & Calvert, 2011), especially for children who are less sports-oriented. Children and adolescents are motivated to play activity-promoting exergames (Staiano, Abraham, & Calvert, 2012b), and exergames induce positive socio-emotional outcomes, including self-efficacy and peer support (Staiano, Abraham, & Calvert, 2013). Schools and youth development programs have begun to incorporate exergames into physical education curriculum (Lieberman, 2006). The current study provides additional support for the potential of incorporating exergames into an applied setting in a way that children enjoy while boosting cognitive abilities.

### 4.2 Future Research

The findings from the current study suggest a number of potential lines of research. First, this study suggested participants improved in EF over five weeks of exergame play, but interventions of longer length that examine if there are lasting effects of game play on EF are needed. Based on the data from the current study, it is not possible to determine how long effects might last (see Staiano et al., 2012a). Second, it is important to explore if type of game play matters. There is currently a wide range of exergames available with varying levels of physical activity and competition. Staiano et al. (2012a) demonstrated that youth in a competitive condition improved EF more than youth in a cooperative condition; therefore, levels of competition in game play should continue to be examined. Third, further research should continue to examine the impact of enjoyment on EF. This study, as well as past research, has demonstrated children are motivated and enjoy playing exergames (Staiano et al., 2012b). Past research has documented that children’s cognition improves when they are performing tasks that they enjoy (e.g., Dias & Harris, 1990; Lepper, Aspinwall, Mumme, & Chabay, 1990; Parker & Lepper, 1992). Thus, future research should continue to examine the relationship between enjoyment of cognitive activities and improvement in cognitive skills.

### 4.3 Limitations

Several limitations in the current study should be considered in interpreting the findings. The first limitation was the lack of an adequate control groups based on the cooperating organization’s desire to have as many children as possible participate in the fitness intervention. Given this limitation, the analyses focused on explaining individual difference factors accounting for changes in EF skills of the participants who played the exergame; however, future research aiming to compare benefits of different kinds of game play or physically-active play should have a control group of adequate size.

A second limitation of the study was lack of direct measurements of heart rate and energy expenditure, which would allow for a better understanding of the relationship between physical exertion and cognitive improvements. To address this limitation, future research should be conducted by objectively measuring physical exertion, such as heart rate or energy expenditure.

A third limitation is that the research took place on-site at a summer camp, rather than in a carefully-controlled laboratory setting. Although the naturalistic camp setting increased the external validity of the findings and is an appropriate context for studying the impact of exergames, some potentially confounding variables could not be controlled, such as varying attendance and retention, differences between group dynamics and playing dyads, the gender distribution of the dyads, and times of day for the game play session. To address these limitations, future research should also be conducted in a controlled setting with a video game that can vary in level of activity.

## 5. Conclusion

Despite the limitations, the current study extends previous research into a relatively poorly understood aspect of children’s media experience: the relationship between cognitive skills and playing exergames. Children who played the exergame over the course of five weeks demonstrated improvements in EF skills. Additionally, children who played for more sessions experienced larger growth in EF. Taken together, these findings have significant implications for using gaming to boost adolescents’ cognitive, and potentially their academic, performance. Future researchers should consider the extent to which different kinds of video game play influence cognitive development. Additionally, practitioners interested in helping adolescents develop these skills should be aware of the potential cognitive benefits of exergames.

## Figures and Tables

**Table 1:** Means (and standard deviations) for demographics by condition

		Experimental	Control groups	

	Total	Highly active exergame	Less active exergame	Non-playing control
Age	13.72 (1.56)	13.20 (1.36)	14.93 (1.59)	11.75 (.96)

% Female	48%	50%	50%	50%

African American, Hispanic/Latino, Biracial	48%, 47%, 5%	49%, 46%, 5%	46%, 47%, 7%	59%, 41%

**Table 2:** Descriptive statistics and correlations between pretest and posttest EF and individual difference factors

Variable	1	2	3	4	5	6	7	8	9
1. Pretest EF	1	.50***	.08	.18	.32*	−.11	−.29*	−.32*	.01
2. Posttest EF	--	1	.08	.35**	.29*	.07	−.30*	−.47**	−.04
3. BMI	--	--	1	.02	.07	.12	−.23	−.31*	−.06
4. Number of exergame sessions	--	--	--	1	.25*	−.08	−.21	−.29	.11
5. Level of achievement	--	--	--	--	1	.08	−.06	−.12	.09
6. Enjoyment	--	--	--	--	--	1	−.01	.06	.13
7. Frustration	--	--	--	--	--	--	1	.69***	.12
8. Boredom	--	--	--	--	--	--	--	1	.01
9. Previous Wii Fit Play	--	--	--	--	--	--	--	--	1
M	5.46	7.86	22.05	3.85	1.74	2.66	2.33	2.04	2.51
SD	4.12	5.06	4.33	1.21	.74	.63	1.56	1.43	1.79

*Note*.

* *p* < .05,

** *p* < .001,

*** *p* < .001

**Table 3:** Multiple regression results for level of exergame sessions on post-test EF scores

Model	Variable	*B*	*SE*	*β*	*t*	*p*	*F*	*R*	*R^2^*	*ΔR^2^*
1	(Constant)	2.36	1.34	−	1.76	.083	18.64*	.48	.23	.23*
	Pretest	.32	.07	.48	4.32	.000				
2	(Constant)	−1.44	1.96	−	−.74	.463	13.46*	.55	.29	.07*
	Pretest	.29	.073	.43	3.98	.000				
	Number of Sessions	1.13	.44	.28	2.58	.012				

* *p* < .05

**Table 4:** Multiple regression results for level of achievement on post-test EF scores

Model	Variable	*B*	*SE*	*β*	*t*	*p*	*F*	*R*	*R^2^*	*ΔR^2^*
1	(Constant)	4.14	1.08	-	3.84	.000	16.42*	.46	.22	.22*
	Pretest	.75	.19	.46	4.05	.000				
2	(Constant)	2.66	1.54	-	1.73	.089	9.21*	.49	.24	.02
	Pretest	.67	.19	.41	3.44	.001				
	Level of achievement	1.08	.81	.16	1.34	.187				

* *p* < .05

**Table 5:** Multiple regression results for level of frustration on post-test EF scores

Model	Variable	*B*	*SE*	*β*	*t*	*p*	*F*	*R*	*R^2^*	*ΔR^2^*
1	(Constant)	3.05	.85	-	3.57	.001	36.95*	.67	.45	.45*
	Pretest	.80	.13	.67	6.08	.000				
2	(Constant)	4.09	1.32	-	3.10	.003	19.04*	.68	.46	.01
	Pretest	.76	.14	.63	5.52	.000				
	Level of frustration	−.35	.34	−.12	−1.0	.307				

* *p* < .05

**Table 6:** Multiple regression results for level of boredom on post-test EF scores

Model	Variable	*B*	*SE*	*β*	*t*	*p*	*F*	*R*	*R^2^*	*ΔR^2^*
1	(Constant)	3.05	.85	-	3.57	.001	36.95*	.67	.45	.45*
	Pretest	.80	.13	.67	6.08	.000				
2	(Constant)	5.55	1.25	-	4.45	.000	24.29*	.72	.52	.07*
	Pretest	.69	.13	.58	5.28	.000				
	Level of boredom	−.95	.36	−.29	−2.63	.012				

* *p* < .05
